# Analytical Determination of the Lipid Fraction of *Nigella sativa* Fatty Oil by GC and NMR Analysis and Evaluation of Its Cytotoxic and Antioxidant Activity

**DOI:** 10.3390/molecules30214300

**Published:** 2025-11-05

**Authors:** Martina Dentato, Antonella Porrello, Elena De Marino, Stefania Ponticelli, Alessia Postiglione, Alessandra Pollice, Maurizio Bruno, Natale Badalamenti, Giuseppe Bazan, Viviana Maresca

**Affiliations:** 1 Department of Biology, University of Naples Federico II, 80126 Naples, Italy; martina.dentato@unina.it (M.D.); elena.demarino@unina.it (E.D.M.); ste.ponticelli@studenti.unina.it (S.P.); alessia.postiglione@unina.it (A.P.); alessandra.pollice@unina.it (A.P.); 2 Department of Biological, Chemical and Pharmaceutical Sciences and Technologies (STEBICEF), University of Palermo, Viale delle Scienze, 90128 Palermo, Italy; antonella.porrello@unipa.it (A.P.); giuseppe.bazan@unipa.it (G.B.); 3 National Biodiversity Future Center (NBFC), 90133 Palermo, Italy; 4 Department of Life Science, Health, and Health Professions, Link Campus University, 00165 Rome, Italy; v.maresca@unilink.it

**Keywords:** Caco-2 cells, DNA damage, GC-MS, HaCaT cells, *Nigella sativa*, NMR, oxidative stress

## Abstract

*Nigella sativa*, or black cumin, is used as a spice in cooking and as a food supplement like seeds or oil for its biological properties, including antioxidant capacity, anti-inflammatory action, and support for the immune system. In the present study, the chemical composition and biological activities of the *Nigella sativa* seeds’ fatty oil (*NS*) were investigated. The analytical composition was carried out by several techniques, such as GC-MS spectrometry and ^1^H- and ^13^C-NMR spectroscopies using appropriate internal standards. The GC-MS analysis highlighted the presence of palmitic and linoleic acid as major compounds. The antioxidant potential was evaluated through the DPPH radical-scavenging assay, and, furthermore, the *NS* effect on intracellular reactive oxygen species (ROS) levels was assessed in HaCaT cells (non-tumorigenic human keratinocytes) under oxidative stress induced by hydrogen peroxide. The cytotoxic and genotoxic profiles were evaluated on Caco-2 cells (human colorectal adenocarcinoma cells) using the CCK-8 viability assay and the Comet assay, respectively. Overall, the results demonstrated that *NS* possessed antioxidant activity, as evidenced by concentration-dependent DPPH radical scavenging and reduced intracellular ROS levels in HaCaT cells under oxidative stress. In Caco-2 colorectal cancer cells, *NS* induced significant cytotoxicity and DNA damage at higher concentrations, suggesting potential genotoxic effects. These findings support the dual role of *NS* as a natural antioxidant and a promising candidate for nutraceutical and dermatological applications, including those targeting oxidative stress-related conditions and cancer.

## 1. Introduction

**Figure 1:**
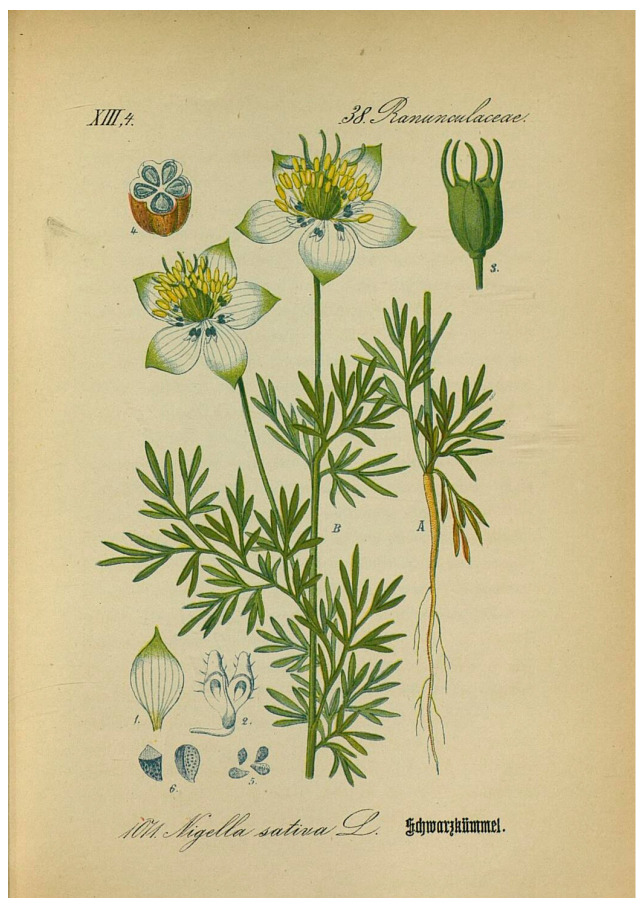
*Nigella sativa* illustrated in Flora von Deutschland by Schlechtendal et al. [3].

*Nigella sativa* L. is an annual therophyte reaching a height of 30 cm or more [1]. The species develops a single, erect stem that tends to branch apically. The stem is cylindrical, striated, and covered with soft hairs between the nodes, and the plant possesses a deep taproot system. The alternate leaves exhibit leaf dimorphism between the basal and cauline types. The basal leaves are linear–spatulate in shape, with petioles that sheath the stem, and are 2–3-pinnatisect, divided into acute and slender segments. The cauline leaves are finely divided into narrower linear segments. The flowers, lacking an involucre of bractiform leaves, are terminal and solitary, and are composed of five sepals that are ovate–acuminate, flat, milky-white, petaloid, and with greenish petals reduced to nectarines. The fruit is an inflated capsule, smooth, ribbed, and composed of five united follicles, each containing numerous small, black seeds (Figure 1) [2]. At maturity, fruits dehisce apically, releasing numerous black, triangular, wrinkled, and aromatic seeds.

*Nigella sativa* has a native Euro-Asiatic distribution range, but due to its ancient cultivation, it has spread as an adventive species, locally naturalized in a wide area extending from Southwestern Asia, across the Mediterranean Basin, to North Africa, and including parts of Southern and Eastern Europe [4].

The seeds of this species, commonly known as black cumin, emit a distinct aromatic odor when rubbed, and have been traditionally used for thousands of years by human populations as a spice and food preservative [5].

The whole plant and its seed oil are renowned for their broad pharmacological spectrum, including antioxidant, anti-inflammatory, immunomodulatory, hepatoprotective, and anticancer properties [6,7]. It has also been used as a medicinal species for the prevention and treatment of various diseases, including diabetes, hypertension, asthma, headache, diarrhea, infections, obesity, back pain, gastrointestinal disorders, inflammation, cough, bronchitis, eczema, fever, dizziness, and flu [8].

Regarding non-volatile compounds, several studies showed the presence of syringic acid, *p*-coumaric acid, thymoquinone, and vitamin E, responsible for the antioxidant properties exhibited by *N. sativa* seeds. In the literature, thymoquinone is often the major bioactive component of *N. sativa* seeds, exerting potent antioxidant and radical-scavenging activity via electron transfer and hydrogen donation mechanisms [9,10].

In addition to thymoquinone, other constituents of the seeds, such as fatty acids, terpenoids, and phenolic compounds, contributed synergistically to biological activities.

With particular regard to fatty oil compounds, *N. sativa* seed oil is characterized by a high content of polyunsaturated fatty acids, predominantly linoleic (55–59%) and oleic (24–28%) acids, followed by palmitic (11–12%) and stearic (2–3%) acids. The lipid fraction is primarily composed of triacyl glycerides, and it is characterized by *ω*-rich components [6].

These constituents have been associated in the literature with multiple pharmacological activities, including cardioprotective, anti-inflammatory, antihypertensive, antioxidant, and immunomodulatory effects. These effects are largely attributed to its diverse phytochemical composition, particularly within the fixed oil, which can contain biologically active compounds, such as palmitic acid, stearic acid, oleic acid, and linoleic acid [11].

Furthermore, *N. sativa* fixed oil has been reported to exert protective roles against metabolic, neurodegenerative, endocrine, and dermatological disorders, highlighting its relevance as a functional and therapeutic lipid source [9,12,13].

The variation in oil composition is influenced by factors such as geographical origin, extraction method, and seed maturity, highlighting the need for chemical standardization and detailed profiling [14].

There are few works demonstrating the antioxidant potential of *N. sativa* fatty oil (*NS*), while a growing body of research supports the antioxidant potential demonstrated through radical-scavenging assays, such as DPPH, ABTS, and FRAP, of essential oils. For example, extracts rich in phenolic content have exhibited IC_50_ values as low as 189.5 µg/mL in DPPH assays, correlating with high levels of flavonoids and monoterpenes [15]. Such antioxidant properties are crucial in the context of oxidative stress, a pathological condition characterized by the overproduction of reactive oxygen species (ROS) that contributes to various chronic diseases, including cancer, cardiovascular disorders, and skin aging [16].

Despite the established antioxidant potential, there is limited evidence regarding the biological behavior of *NS* in specific cellular models, especially human keratinocytes (HaCaT) and intestinal epithelial cells (Caco-2), which are highly relevant for dermatological and nutraceutical applications, respectively. Moreover, safety concerns such as cytotoxicity and genotoxicity remain underexplored, especially in relation to dose-dependence and ROS modulation under stress conditions [17].

Therefore, the present study aims to bridge this knowledge gap by the following: (i) clear chemical characterization of *Nigella sativa* fatty oil using gas chromatography–mass spectrometry (GC-MS) and ^1^H- and ^13^C-NMR spectroscopy; (ii) evaluating its antioxidant activity through the DPPH free radical-scavenging assay; (iii) investigating its intracellular antioxidant role by measuring ROS levels in HaCaT cells under oxidative stress induced by hydrogen peroxide; (iv) assessing its cytotoxic and genotoxic effects in Caco-2 human colorectal adenocarcinoma cells using the CCK-8 viability assay and Comet assay. These studies will support its potential application as a natural antioxidant agent in nutraceutical formulations or protective cosmeceuticals, particularly in preventing oxidative damage in epithelial tissues.

## 2. Results and Discussion

### 2.1. Chemical Analysis of NS

*NS* was obtained by static petroleum ether extraction, starting from a commercial sample of mature *Nigella sativa* seeds with a yield of 13.2% *w*/*w*.

**Figure 2:**
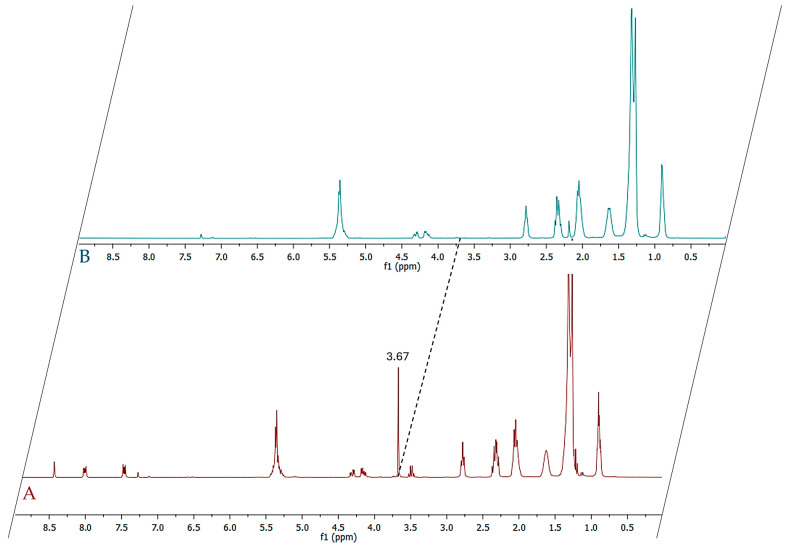
(**A**) ^1^H-NMR spectrum of methylated *NS* fatty oil; (**B**) ^1^H-NMR spectrum of original *NS* fatty oil.

First, the percentage of free fatty acids (FFA) in *NS* was determined. To obtain this data, the method proposed by Satyarthi et al. [18] was used. It is based on integrations of the triplet signal, in ^1^H-NMR spectra (Figure 2A,B), corresponding to methylene protons directly adjacent to the COOH group (at 2.36 ppm) and the same signal of esterified fatty acids at 2.34 ppm. Although the presence of these chemical structures can be highlighted experimentally, due to their overlapping, these signals cannot be used for a correct quantification.

Alternatively, the ^13^C-NMR technique can be used for the determination of the free acidity of vegetable oils by quantifying the percentage of FFA with respect to the total fatty acids. In fact, the free carbonyl signals resonate between 176 and 180 ppm, while the esterified carbonyls in the region resonate between 171 and 174 ppm [19]. By comparing the area of the signals of the two regions, the free acidity (% mole fraction) can be easily calculated.

However, attention must be paid in order to obtain an accurate quantification, since carboxyl and carbonyl carbon atoms have significantly longer relaxation times than protons. To obtain quantitative data, the relaxation issue can be bypassed by running the ^13^C-NMR spectra with a sufficiently long relaxation delay [20].

**Figure 3:**
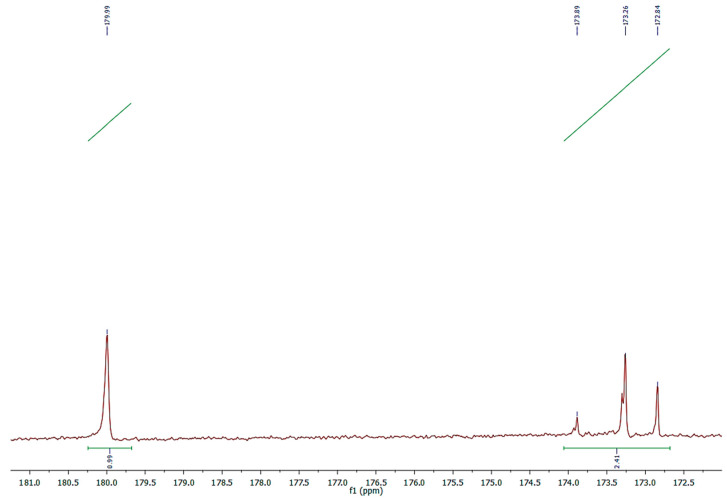
Expanded ^13^C-NMR spectrum of carbonyl carbons in free and esterified acids of *NS*.

The ^13^C-NMR of *NS* clearly identifies two groups of signals in the area of carbonyl carbons (Figure 3). The first one, centered at 179.99 ppm, is relative to the free carboxyl acid, whereas the second one, occurring between 172 and 174 ppm, concerns the carbonyl of glycerol esters.

The percentage of FFA, calculated by the formula presented in the *3.5* paragraph that uses integrations of the signals in the area of 179.99 ppm and those relative to all the carbonyls in the region 172–180 ppm, was 29.5% of the total fatty profile.

The chemical composition of FFA was determined using the GC–MS analysis (Table 1).

The chemical profile of *NS* was fully characterized, and six fatty acids were clearly identified. Among them, linoleic acid (57.2%) was the most abundant, followed by oleic acid (21.3%). In addition to these two unsaturated fatty acids, *NS* also contained a moderate amount of palmitic acid (12.3%), stearic acid (3.9%), eicosadienoic acid (4.6%), and gondoic acid (0.7%), completing the free fatty acid profile.

**Figure 4:**
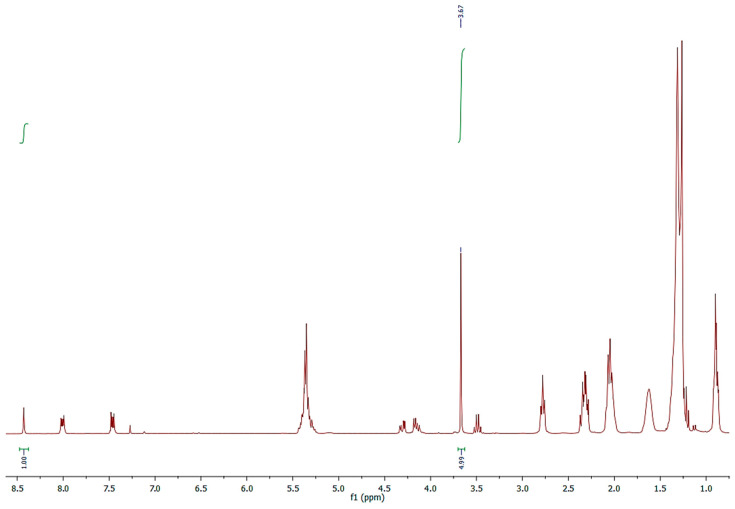
^1^H-NMR spectrum of methylated *NS* acquired in CDCl_3_-*d* with anthracene as internal standard.

The validation of the quantitative composition of the FFA profile of *NS* was obtained, however, via a novel method, by running the ^1^H-NMR spectrum of methylated *NS* with an internal standard (Figure 4).

An exactly weighted quantity of *NS* was completely methylated by means of diazomethane, converting all FFA into methyl esters, dissolving in CDCl_3_-*d*, and adding anthracene as an internal standard (Figure 4).

The micromoles of the FFAs were determined by integrating the signals of the two protons, H_9_ and H_10_, of anthracene at 8.43 ppm and the OC*H_3_* signals of the methylated fatty acids at 3.67 ppm, applying the formula shown in Section 3.6.

The result (Table 1), 0.089 *mmol*_(_*_FFA_*_)_, compared with the relative percentage of FFA determined using the GC-MS, allowed for the exact quantification (mg/100 mg of *NS*) of the single fatty acid and of the total FFA amount (26.78 mg). The result obtained via this method was validated and confirmed by determining, in parallel, the free acidity of vegetable oils by ISO 729:1988 standardized titration [22].

In addition, an extracted quantity of *NS* (500 mg) was subjected to transesterification in order to evaluate the fatty acid composition of the glycerid portion. Following derivatization, the total fatty acid profile was characterized using GC–MS, with complete identification of the detected constituents (Table 2). Fourteen fatty acids were identified. Despite the broader qualitative profile, linoleic acid remained the predominant component, representing 64.9% of the total composition. In contrast to the FFA profile, palmitic acid (15.2%) emerged as the second most abundant fatty acid, followed by oleic acid (8.0%). Stearic acid and eicosadienoic acid were present at lower proportions (5.5 and 4.3%, respectively). The remaining fatty acids occurred only in trace amounts, ranging between 0.1% and 0.5%.

The chemical composition obtained is in line with what has been reported in the literature; indeed, the lipid profile of *NS* from different geographical origins has been extensively studied [23,24,25,26,27,28], and in most cases, linoleic acid is reported as the most abundant fatty acid, although the percentages may vary depending on factors such as the extraction method and solvent used, as well as the ripening stage of the seeds.

Indeed, although studies have shown that the extraction technique does not significantly affect the lipid composition of the oil, it has been observed that other parameters, such as free acidity, yield, and oxidative stability, vary depending on the extraction method used. In particular, the Soxhlet extraction technique has been reported to offer notable advantages, increasing the oil yield by approximately 4–6% compared with other extraction methods. Moreover, the oil obtained through Soxhlet extraction exhibits a higher proportion of unsaturated fatty acids, indicating improved nutritional quality [29].

However, a noteworthy case is a study on wild *N. sativa* seeds collected in Yemen [30]. In this case, the data show that, unlike most of the findings reported in the literature for seeds of this species, oleic acid is the main constituent of the oil (20.6–21.5%), while linoleic acid is present in very limited amounts.

The presence of other fatty acids detected in trace amounts in *Nigella sativa* seed oil after transesterification, such as behenic, margaric, myristic, and eicosenoic acids, has been reported for other samples [31,32]. In particular, Atta et al. [31] reported a composition characterized by 9.8% myristic acid, while in the sample analyzed in this study, it was present only at 0.5%. Similarly, margaric acid, an uncommon 17-carbon fatty acid compound in vegetable oils, was found in amounts of 10.3% in an oil obtained from commercial Indian *Nigella* seeds [27].

### 2.2. DPPH Radical-Scavenging Assay

**Figure 5:**
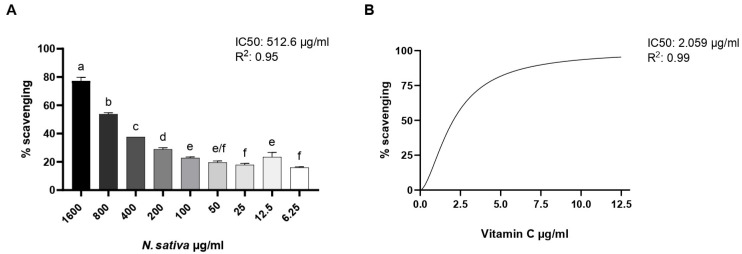
(**A**) DPPH radical-scavenging activity of NS at different concentrations (6.25–1600 µg/mL). (**B**) Ascorbic acid was used as a positive control for test efficacy. Data are expressed as mean ± SD (n = 3). Different letters represent the significance between different concentrations. One-way ANOVA followed by Tukey’s post hoc test for multiple comparisons.

The antioxidant potential of *NS* was evaluated using the DPPH free radical-scavenging assay. As shown in Figure 5, the pure oil exhibited a dose-dependent increase in radical-scavenging activity, with higher concentrations leading to greater inhibition of DPPH absorbance. Maximum activity was observed at 1600 µg/mL, with a gradual decline as concentrations decreased. The oil maintained over 40% scavenging activity down to 200 µg/mL, while lower concentrations (≤25 µg/mL) showed a limited effect, comparable to the negative control. The concentration inhibiting 50% of DPPH was found to be 512.6 µg/mL. Ascorbic acid, used as the positive control, displayed the highest radical-scavenging effect, validating the assay.

Mraihi et al. [33] demonstrated that hexane extracts of *N. sativa* from Tunisia, Libya, and Saudi Arabia have EC_50_ values of 507.50, 528.00, and 422.04 μg/mL, respectively. These values are consistent with our results, as both the EC_50_ and IC_50_ in the DPPH test indicate the concentration of the tested extract capable of reducing the DPPH free radical.

### 2.3. Intracellular ROS Assay in HaCaT Cells

**Figure 6:**
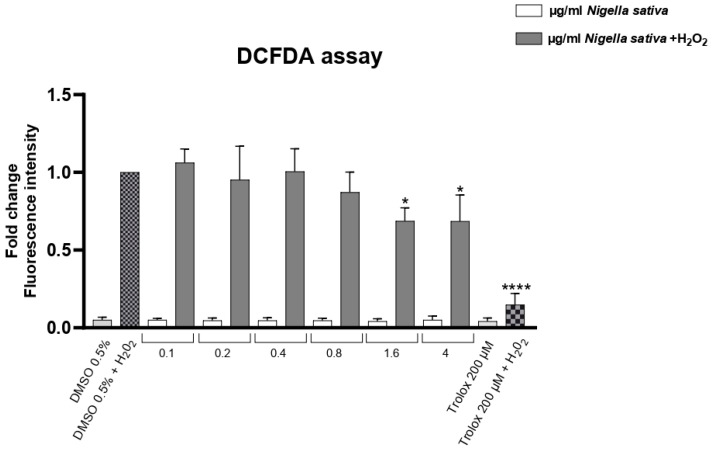
Effect of *NS* on intracellular ROS levels in HaCaT cells under oxidative stress (+H_2_O_2_). Data are expressed as mean ± SD (n = 3). * *p* < 0.05, **** *p* < 0.0001 vs. untreated control (one-way ANOVA followed by Dunnett’s post hoc test).

HaCaT keratinocytes (CLS Cell Lines Service GmbH, Eppelheim, Germany) were exposed to hydrogen peroxide (H_2_O_2_) to induce oxidative stress after being treated with increasing concentrations of *NS* (Figure 6). Intracellular reactive oxygen species (ROS) levels were measured and are represented as relative fluorescence intensity. Basal ROS levels were found to be within physiological ranges in all conditions, ruling out the presence of pre-existing oxidative stress. H_2_O_2_ treatment markedly increased ROS levels compared to the untreated control, represented by DMSO0.5%. Treatment with *NS* led to a progressive, dose-dependent decrease in ROS levels, with the highest concentration showing the most significant antioxidant effect. A total of 200 µM of Trolox was used as a positive control.

### 2.4. Cytotoxicity Assay on Caco-2 Cells (CCK-8)

**Figure 7:**
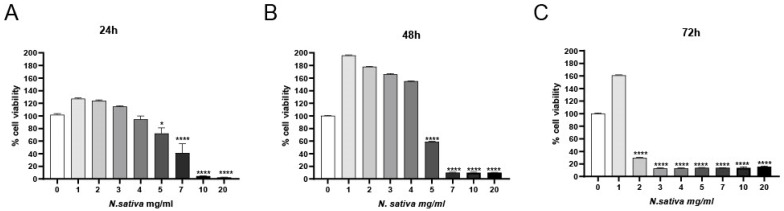
Cytotoxic effects of *NS* on Caco-2 cells assessed via CCK-8 assay at (**A**) 24 h, (**B**) 48 h, and (**C**) 72 h. Data are presented as mean ± SD (n = 3). * *p* < 0.05, **** *p* < 0.0001 vs. untreated control (one-way ANOVA followed by Dunnett’s post hoc test).

The cytotoxic activity of *NS* was evaluated in human colorectal adenocarcinoma cells (Caco-2; ATCC, Manassas, VA, USA) using the CCK-8 viability assay after 24, 48, and 72 h of treatment with increasing concentrations of the oil (0–20 mg/mL). As shown in Figure 7, a clear time- and dose-dependent reduction in cell viability was observed. At 24 h (Figure 7A), significant cytotoxic effects emerged at concentrations ≥ 3 mg/mL (*p* < 0.05), with a more pronounced reduction at 5–20 mg/mL (*p* < 0.0001). The cytotoxic effect intensified over time, as evidenced by further decreases in viability at 48 h (Figure 7B) and 72 h (Figure 7C), with nearly complete loss of viability at the highest concentrations.

### 2.5. Comet Assay in Caco-2 Cells

**Figure 8:**
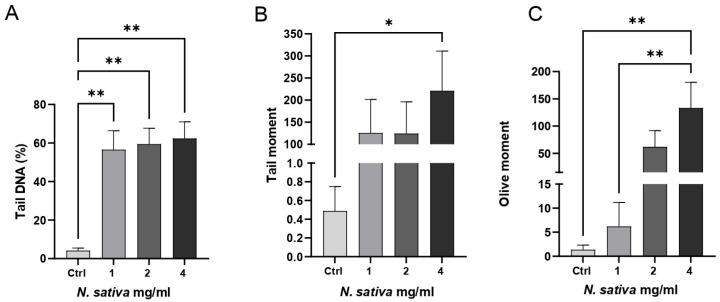
Genotoxicity assessment of *NS* in Caco-2 cells using the alkaline Comet assay. Cells were treated with increasing concentrations of the oil (1, 2, and 4 mg/mL) for 24 h. (**A**) The percentage of tail DNA, (**B**) tail moment, and (**C**) olive tail moment were quantified to evaluate DNA damage. Data are presented as mean ± SD (n = 4). * *p* < 0.05, ** *p* < 0.01 vs. untreated control (one-way ANOVA followed by Dunnett’s post hoc test).

The genotoxic potential of *NS* was assessed in Caco-2 cells using the alkaline Comet assay. As shown in Figure 8, treatment with increasing concentrations of the oil (1, 2, and 4 mg/mL) for 24 h induced a dose-dependent increase in DNA strand breaks, as indicated by significant alterations in key Comet parameters.

Specifically, the percentage of tail DNA increased significantly at both 2 and 4 mg/mL compared to the untreated control (*p* < 0.01), suggesting elevated levels of DNA fragmentation (Figure 8A). The tail moment also rose with increasing oil concentrations and reached statistical significance at 4 mg/mL (*p* < 0.05), reflecting both the extent and distribution of damaged DNA within the nucleus (Figure 8B). Similarly, the olive tail moment was markedly elevated at 2 and 4 mg/mL (*p* < 0.01), further confirming the genotoxic effect (Figure 8C).

These findings indicate that *NS* can induce moderate but significant genotoxic stress in Caco-2 cells at higher concentrations. Given that these cells represent a human colorectal adenocarcinoma model, such DNA-damaging activity may be considered therapeutically advantageous, reflecting a potential pro-apoptotic or antiproliferative mechanism against malignant cells. The absence of significant genotoxic effects at 1 mg/mL suggests the existence of a concentration threshold for DNA damage induction. Fluorescence microscopy images (Olympus BX53, Olympus Corporation, Tokyo, Japan) of the Comet assay are shown in Supplementary Materials (Figure S1), illustrating control cells and treated cells, with a progressive increase in comet tail formation observed at higher concentrations.

The present study highlights that *NS*, dominated by linoleic acid together with oleic and palmitic acids, exhibited a dual biological activity: antioxidant protection in non-tumoral keratinocytes and cytotoxic as well as genotoxic effects in colorectal carcinoma cells. In HaCaT cells, the oil reduced hydrogen peroxide–induced intracellular ROS in a clear dose-dependent manner. This observation is consistent with previous reports that fatty acid–rich plant oils, particularly those abundant in linoleic acid, can attenuate oxidative stress and support cellular homeostasis. Manosalva et al. [34] showed, for example, that linoleic acid alone was sufficient to decrease ROS levels and reprogram metabolic responses in keratinocytes exposed to UVB irradiation, which is in line with the antioxidant effect observed here. Similarly, Sultan et al. [35] demonstrated that dietary administration of *NS* enhanced the activities of glutathione-related enzymes in vivo, supporting the capacity of the lipid fraction to modulate redox balance at the systemic level.

The cytotoxic effects observed in Caco-2 cells are also consistent with other studies that investigated non-volatile lipid fractions of *N. sativa*. Mahmoud & Torchilin [36] reported that lipid extracts decreased MCF-7 breast cancer cell viability with an LC_50_ in the milligram per milliliter range, a concentration range comparable to the effects reported here in colon carcinoma cells. These results suggest that the cytotoxicity is not restricted to a single tumor type and can be exerted by the lipid fraction of the seed itself. Comparable effects have also been described for other plant fatty oils rich in unsaturated fatty acids. For instance, vegetable oils high in linoleic and oleic acids have been shown to impair the proliferation and migration of tumor cells, often through mechanisms linked to oxidative imbalance, lipid peroxidation, and mitochondrial dysfunction [37].

Biologically, *NS* demonstrated pronounced cytotoxic effects on Caco-2 colorectal adenocarcinoma cells. The oil induced a robust time- and dose-dependent decrease in cell viability, with significant effects at higher doses and prolonged exposure. The Comet assay analysis confirmed a concentration-dependent induction of DNA damage (increases in % tail DNA, tail moment, olive tail moment) at mid-to-high doses, whereas no significant genotoxicity was observed at the lowest tested dose. These findings suggest a threshold effect, with lower concentrations possibly exerting neutral or antioxidant effects, and higher concentrations triggering cytotoxicity and DNA fragmentation.

The observed potency of *NS* (in the mg·mL^−1^ range) aligns with patterns reported for other vegetable fatty oils, which often require relatively high mass concentrations to exhibit cytotoxicity in vitro. For example, açaí seed oil produced cytotoxic effects in Caco-2 and HCT-116 cells (in the mg/mL range), associated with apoptosis induction [38]. These observations support the plausibility of the cytotoxic concentrations we report.

What emerges from our data is an apparent functional duality: in non-tumoral keratinocytes, the oil provides protection against oxidative stress, whereas in tumor cells, it exerts damaging effects on viability and DNA integrity. This contrast may be explained by the different metabolic and oxidative states of normal versus malignant cells, with cancer cells being more vulnerable to lipid peroxidation and ROS imbalance. At moderate concentrations, the unsaturated fatty acids present in the oil may reinforce cellular antioxidant defenses, while at higher concentrations, their susceptibility to peroxidation may generate reactive by-products capable of damaging tumor cell DNA. This hypothesis aligns with reports on other fatty acid-rich plant oils, which often display protective effects in normal cells and cytotoxic effects in cancer cells, though the selectivity window can vary considerably [39].

Taken together, our findings indicate that *NS* is biologically active independently of volatile constituents and that its activity is largely attributable to its unsaturated fatty acid composition. The concentrations required are similar to those reported for other seed oils tested in vitro, suggesting that while the potency is moderate, the dual effect, antioxidant in normal cells and cytotoxic in malignant ones, could be exploited in nutraceutical or dermatological contexts. Further research should clarify the mechanisms underlying these effects, establish whether genotoxicity is selective for tumor cells, and explore formulation strategies to enhance activity and safety.

## 3. Materials and Methods

### 3.1. Extraction Procedure

Seeds (50 g) of commercial *Nigella sativa *(Shaman Spices & Food Products LLP, Kurali Taluka: Karjan, Dist: Vadodara-391240, India) were reduced to a fine powder using a Mulinex blender type AR110830 (De’Longhi Appliances s.r.l—Commercial Division Ariete, Firenze, Italy) and extracted three times with petroleum ether (400 mL) under stirring at room temperature for 72 h. The solvent was evaporated at 40 °C using a Buchi rotavapor R-200 (BUCHI Italia s.r.l., Via Galileo Galilei 34, 20007 Cornaredo, Italy) to give 6.6 g of *NS* (yield 13.2%). The resulting oil was kept away from light and at a low temperature. The petrolium ether (Cas-No. 64742-49-0) was purchased from Carlo Erba (CARLO ERBA Reagents srl, Via Raffaele Merendi 22, 20007 Cornaredo, Italy).

### 3.2. Sample Preparation

An exact quantity (92 mg for ^1^H-NMR analysis and 100 mg for GC-MS analysis) of *NS* was treated with an ethereal solution of CH_2_N_2_, which was added dropwise at room temperature until a persistent pale-yellow color developed. The reaction has been checked via TLC and GC-MS analysis until the disappearance of the free fatty acid signal. Excess CH_2_N_2_ was removed by gentle warming under a fume hood, and the solvent was subsequently evaporated. For ^1^H-NMR analysis, 92.0 mg of methylated fatty oil was dissolved in CDCl_3_-*d* and added with 5.0 mg of anthracene, which was used as an internal standard.

In addition, 500 mg of *NS* was reacted with 0.7 mL of 10 M KOH and 5.3 mL of MeOH, and the mixture was allowed to react under stirring for 90 min at 55 °C. After cooling to room temperature, 0.58 mL of a 72% H_2_SO_4_ solution was added. The sample was stirred until the formation of a potassium sulfate precipitate was observed and subsequently reheated at 55 °C for 90 min. Finally, following partitioning with hexane (3 mL), the organic phase was recovered, dried over Na_2_SO_4_, and subjected to methylation, using the same procedure previously described.

TLC and all chemicals, such as anthracenes (Cas-No. 120-12-7), CH_2_N_2_ (Cas-No. 80-11-5), KOH (Cas-No. 1310-58-3), K_2_SO_4_ (Cas-No. 7778-80-5), and Na_2_SO_4_ (Cas-No. 7757-82-6), were purchased from Sigma Aldrich (Merk Life Science S.r.l., Via Monte Rosa 93, 20149 Milan, Italy) and used without further purification; CDCl_3_-*d* (Cas-No. 865-49-6), ether (Cas-No. 60-29-7), MeOH (Cas-No. 67-56-1), hexane (Cas-No. 110-54-3), and H_2_SO_4_ (Cas-No. 7664-93-9) were all of special grade (Carlo Erba, Cornaredo, Italy).

### 3.3. Gas Chromatography–Mass Spectrometry (GC-MS) Analysis

Diazomethylated samples (100 mg) were analyzed using gas chromatography combined with mass spectrometry (GC-MS), using a Shimadzu QP 2010 plus equipped with an AOC-20ia autoinjector (SHIMADZU CORPORATION, Nishinokyo Kuwabara-cho, Nakagyo-ku, Kyoto 604-8511, Japan) gas chromatograph equipped with a non-polar capillary column (DB-5MS) 30 m × 0.25 mm i.d., film thickness 0.25 μm, and a data processor. The oven program was as follows: temperature increase at 40 °C for 5 min, at a rate of 2 °C/min up to 260 °C, then isothermal for 20 min. Helium was used as a carrier gas (1 mL min-1). The injector and detector temperatures were set at 250 °C and 290 °C, respectively. A total of 1 μL of solution (3% sample/hexane *v*/*v*) was injected with split mode 1.0; MS range 40–600. The analyses were performed in triplicate, and the results in Table 1 and Table 2 are expressed as the average of three measurements ± standard deviation. The MS settings were as follows: ionization voltage, 70 eV; electron multiplier energy, 2000 V; transfer line temperature, 295 °C; solvent delay, 3 min. Linear Retention Indices (LRI) were determined by using retention times of *n*-alkanes (C_8_-C_40_), and the peaks were identified by comparison with mass spectra and by comparison of their relative retention indices with WILEY275, NIST 17, ADAMS, and FFNSC2.

### 3.4. Nuclear Magnetic Resonance (NMR) Analysis

The NMR spectra were recorded on a Bruker Avance II instrument (Bruker Italia srl, Viale Vincenzo Lancetti, 43, 20158 Milan, Italy).

^1^H-NMR spectra were acquired at room temperature using a BBO (probe model) probe head set to 400 MHz. The proton 90-degree pulse width for the 400 MHz was 7.75 μs. Relaxation delays were limited to 2 s (short), and the number of scans was 16. Chemical shifts (δ) were indirectly referred to tetramethylsilane using residual solvent signals. Deuterated solvents, such as CDCl_3_-d, were used for the solubilization of the various samples. A residual CDCl_3_-d solvent signal of 7.27 ppm was used as reference in ^1^H-NMR spectra.

For the acquisition of ^13^C NMR spectra, considering the long relaxation times, decoupled pulse programs were used. ^13^C-NMR spectra were acquired at room temperature using a BBO (probe model) probe head set to 100 MHz. Relaxation delays were limited to 30 s (long) for carbon spectra acquisition; the total number of data points collected was 64k; and the number of scans was 8000. Baseline correction was performed using the 5th-order polynomial functions from −10 to 240 ppm range. When processing data, line broadening (LB) was set to 0.0, the size of the real spectrum (SI) was set to 131,072 (128 K, 2 × TD) data points, and all mathematical fits employed during the deconvolution procedure were 100% Lorentzian in nature. If LB is set to 0.0 and SI is not 2 × TD, peak widths at half-height of 0.0 may cause the deconvolution procedure to terminate prematurely. A residual CDCl_3_-*d* solvent signal of 77.00 ppm was used as reference in ^13^C-NMR spectra.

### 3.5. Determination of Free Fatty Acids (FFA) Percentage

The percentage of FFA was determined by applying the following formula:

$$\%\;FFA\;=\frac{I_{{CO}_{FFA}}\;}{I_{total\;{CO}_{FFA}+\;{CO}_{esters}}}\times 100$$

where $I_{{CO}_{FFA}}$ is the integral of the signals in the area of 179.99 ppm, and $I_{total\;{CO}_{FFA}+\;{CO}_{esters}}$ is the integral of the area relative to all the carbonyls in the region 172–180 ppm.

### 3.6. Quantification of Free Fatty Acids

The micromoles of *FFA* were determined by integrating the signals of the two protons, H-9 and H-10, of anthracene at 8.43 ppm and the OC*H_3_* signals of the methylated fatty acids at 3.67 ppm, applying the following formula:

$${mmol}_{\left(FFA\right)}=\frac{{mmol}_{\left(A\right)}\times I_{\left(OMeFFA\right)}\;\times \;2\;}{\;I_{\left(H_{9},H_{10}\;A\right)}\;\times \;3}$$

where ${mmol}_{\left(FFA\right)}$ refers to the micromoles of free fatty acids, ${mmol}_{\left(A\right)}$ refers to the millimoles of anthracene, $\;I_{\left(H_{9},H_{10}\;A\right)}$ is the integral of the two protons, $H_{9}$ and $H_{10}$, of anthracene at 8.43 ppm, $I_{\left(OMeFFA\right)}$ is the integral of the three protons of the methoxy groups at 3.67 ppm, and 2/3 is the ratio considering the number of protons.

### 3.7. DPPH Radical-Scavenging

The antioxidant activity of *NS* was assessed using the 2,2-diphenyl-1-picrylhydrazyl (DPPH) radical-scavenging assay, following a slightly modified version of the method described by Brand-Williams et al. [40]. A DPPH solution was prepared by dissolving 3.94 mg of DPPH in 50 mL of absolute ethanol, yielding a final concentration of 0.2 mM. The solution was stored in the dark at room temperature and used within 24 h of preparation.

For the assay, 100 µL of the 0.2 mM DPPH solution was added to each well of a 96-well microplate, followed by 100 µL of the oil sample previously diluted in ethanol at the following concentrations: 1600, 800, 400, 200, 100, 50, 25, 12.5, and 6.25 µg/mL

Negative control wells contained 100 µL of ethanol instead of the sample. Sample blanks (to account for auto-absorbance) were prepared by mixing 100 µL of the diluted sample with 100 µL of ethanol, without the addition of DPPH. Ascorbic acid was used as a positive control.

All measurements were performed in technical quadruplicate. The microplate was incubated in the dark at room temperature for 30 min. After incubation, the absorbance was read at 517 nm using a microplate reader (BioTeck Instruments, Inc., Winooski, VT, USA).

The percentage of DPPH radical scavenging activity was calculated using the following equation:

$$\%\;Scavenging=\;\left[\frac{Acontrol-(Asample-Ablank)}{Acontrol}\right]\times \;100\;$$

### 3.8. Determination of IC_50_ Value

The half-maximal inhibitory concentration (IC_50_) of *NS* was determined from the DPPH radical-scavenging data. The percentage of scavenging activity for each tested concentration was plotted against the logarithm of the concentration using GraphPad Prism (v.10, GraphPad Software, San Diego, CA, USA).

A nonlinear regression model (four-parameter logistic curve) was used to fit the dose–response data and calculate the IC_50_ value, defined as the concentration of the sample required to inhibit 50% of DPPH radicals. All calculations were based on data obtained from four independent technical replicates per concentration.

For comparative purposes, the IC_50_ of the reference antioxidant compound, ascorbic acid, was also determined under the same experimental conditions.

### 3.9. Intracellular ROS Assay

The intracellular antioxidant effect of NS was evaluated in HaCaT cells (immortalized human keratinocytes) exposed to oxidative stress induced by hydrogen peroxide (H_2_O_2_). Cells were cultured in Dulbecco’s Modified Eagle Medium (DMEM) according to Battah et al. [41], supplemented with 10% fetal bovine serum (FBS), 1% penicillin–streptomycin, and maintained at 37 °C in a humidified atmosphere with 5% CO_2_.

HaCaT cells were seeded into black 96-well plates at a density of 4 × 10^4^ cells/well and allowed to adhere overnight. Cells were pretreated with different concentrations of *NS* (0,1 to 4 µg/mL) diluted in culture medium for 3 h, followed by exposure to 200 µM H_2_O_2_ for 1 h and 30 min to induce oxidative stress. The untreated control was represented by cells treated with 0,5% of DMSO.

Before inducing H_2_O_2_ oxidative stress, basal ROS levels were measured for each concentration in order to rule out pre-existing oxidative stress conditions.

After treatment, intracellular ROS levels were measured using 2′,7′-dichlorodihydrofluorescein diacetate (DCFH-DA, Sigma-Aldrich, St. Louis, MO, USA). Cells were incubated with 10 µM DCFH-DA for 45 min at 37 °C in the dark, washed with PBS, and fluorescence intensity was measured using a microplate reader (excitation: 485 nm, emission: 530 nm). Results were expressed as the percentage of ROS generation relative to H_2_O_2_-treated controls.

### 3.10. Cytotoxicity Assay

The cytotoxic activity of *NS* was evaluated in human colorectal adenocarcinoma (Caco-2) cells using the Cell Counting Kit-8 (Enhanced Cell Counting Kit-8 WST-8/CCK8) (Elabscience, Houston, TX, USA). Caco-2 cells were seeded into 96-well plates at a density of 3 × 10^3^ cells/well in 100 μL of complete Dulbecco’s Modified Eagle Medium (DMEM) and incubated at 37 °C in a 5% CO_2_ humidified atmosphere for 24 h to allow cell adherence.

Cells were treated with increasing concentrations of the oil (0 to 20 mg/mL) for 24, 48, and 72 h. The negative control was represented by 0 mg/mL treatment (0.5% DMSO). After incubation, 10 µL of CCK-8 reagent was added to each well, and plates were incubated for 2 h at 37 °C. Absorbance was measured at 450 nm using a microplate reader (Bio-Rad, Hercules, CA, USA).

Cell viability was calculated as a percentage of the absorbance relative to untreated control wells. All experiments were performed in quadruplicate. The IC_50_ value was determined using nonlinear regression analysis.

### 3.11. Genotoxicity Assessment by Comet Assay

DNA damage induced by *NS* was assessed in Caco-2 cells using the comet assay, following the protocol by Singh et al. [42], with minor modifications according to Chianese et al. [43], and 4 × 10^5^ Caco-2 cells were seeded in 60 mm dishes. After 24 h, cells were treated with various concentrations of the oil (1–2 and 4 mg/mL) for 24 h, harvested, and embedded in 1% low-melting agarose on microscope slides precoated with 1% normal-melting agarose.

After lysis (2.5 M NaCl, 100 mM EDTA, 10 mM Tris, 1% Triton X-100, pH 10) for 1 h at 4 °C, slides were placed in electrophoresis buffer (300 mM NaOH, 1 mM EDTA, pH > 13) for 20 min to allow DNA unwinding. Electrophoresis was performed at 25 V and 300 mA for 20 min. Slides were neutralized with Tris buffer (0.4 M, pH 7.5) and stained with ethidium bromide (5 µg/mL) (Sigma-Aldrich, Darmstadt, Germany).

A fluorescence microscope was used to examine the slides, analyzing a minimum of 50 randomly selected nuclei from each slide and avoiding overlapping figures. A computerized image-analysis system (CometScore, TriTek Corp, Sumerduck, VA, USA) was employed. Twenty-five nuclei were scored per slide, three slides were evaluated per treatment, and each treatment was repeated at least twice. From the repeated experiments, DNA in the tail, tail moment, and olive moment from each slide were calculated.

### 3.12. Statistical Analysis

All activities were analyzed using one-way analysis of variance (ANOVA), followed by either Tukey’s multiple comparison post hoc test or Dunnett’s post hoc test.

Data were collected from three biological replicates for each measurement. In Figure 4, Figure 6, Figure 7 and Figure 8, activities were analyzed using the one-way analysis of variance (ANOVA), followed by Dunnett’s post hoc test. Values are presented as mean ± SD, and significant differences are indicated by asterisks (* *p* < 0.05, ** *p* < 0.01, **** *p* < 0.0001). In Figure 5, activities were analyzed using one-way ANOVA followed by Tukey’s multiple comparison post hoc test. Values are presented as mean ± SD, and groups not sharing the same letter are significantly different at *p* < 0.05. All data were analyzed using GraphPad Prism version 9.0 (GraphPad Software, San Diego, CA, USA).

## 4. Conclusions

In this study, NMR and GC-MS techniques were used to characterize the fatty acid profile of fatty oil extracted from commercial *Nigella sativa* (*NS*) seeds. The chemical composition in terms of free fatty acids and total fatty acids of *NS* was characterized by gas chromatography, with linoleic acid as the predominant component. ^13^C-NMR spectroscopy study on NS further quantified the free acidity of the sample, which amounts to 29.5%. Furthermore, the combination of ^1^H-NMR analysis with the use of a standard allowed for a clear quantitative characterization of the free fatty acids in *NS*.

The antioxidant and cytotoxic activities of *NS* were also evaluated. The results highlight the dual functionality of *NS* as both a natural antioxidant and a cytotoxic agent against colorectal cancer cells. Though linoleic and oleic acids may underlie much of the antioxidant capacity, the observed cytotoxicity and DNA damage suggest that the oil’s full matrix contributes to the net bioactivity. Variability in oil composition across origins and processing methods further underscores the need for standardized extraction and chemical characterization. Further in vivo investigations and mechanistic studies are warranted to delineate safe and efficacious applications in oxidative-stress–related conditions and colorectal cancer models.

## Figures and Tables

**Table 1:** Relative and quantitative compositions of FFA (free fatty acids) in *Nigella sativa* fatty oil.

No.	Fatty Acids	LRI ^a^	LRI ^b^	PM_FFA_ ^c^	Area (%)	mg/100 mg ^d^
1	Palmitic acid (C16:0)	1927	1927	256	12.18 ± 0.53	3.02
2	Stearic acid (C18:0)	2143	2128	284	3.78 ± 0.12	1.06
3	Oleic acid (C18:1)	2115	2105	282	21.32 ± 1.01	5.76
4	Linoleic acid (C18:2)	2103	2099	280	57.16 ± 2.98	15.38
5	Gondoic acid (C20:1)	2290	2321	306	0.73 ± 0.02	0.20
6	Eicosadienoic acid (C20:2)	2283	2285	308	4.49 ± 0.19	1.36
	**Total**				**99.66 ± 4.85**	**26.78**

^a^ Experimental Linear Retention Indices (LRIs) on DB-5MS apolar column; ^b^ Linear Retention Indices based on literature [21]; ^c^ PM_FFA_ is free fatty acid molecular weight; ^d^ mg of FFA in 100 mg of *NS*.

**Table 2:** Chemical composition (%) of fatty acids profile in Nigella sativa fatty oil after transesterification reaction.

No.	Fatty Acids	LRI ^a^	LRI ^b^	Area (%)
1	Myristic acid (C14:0)	1722	1726	0.51 ± 0.03
2	Pentadecanoic acid (C15:0)	1822	1820	0.10 ± 0.01
3	Palmitic acid (C16:0)	1927	1927	15.21 ± 0.58
4	Palmitoleic acid (C16:1)	1899	1896	0.52 ± 0.02
5	Margaric acid (C17:0)	2014	2028	0.22 ± 0.01
6	Stearic acid (C18:0)	2143	2128	5.55 ± 0.34
7	Oleic acid (C18:1)	2115	2105	8.03 ± 0.39
8	Ricinoleic acid (C18:1)	2269	2275	0.30 ± 0.01
9	Linoleic acid (C18:2)	2103	2099	64.72 ± 4.12
10	Arachidic acid (C20:0)	2328	2332	0.29 ± 0.01
11	Gondoic acid (C20:1)	2290	2321	0.10 ± 0.00
12	Eicosadienoic acid (C20:2)	2283	2285	4.20 ± 0.26
13	Behenic acid (C22:0)	2527	2527	0.09 ± 0.00
14	Lignoceric acid (C24:0)	2727	2729	0.10 ± 0.00
	**Total**			**99.91 ± 5.78**

^a^ Experimental Linear Retention Indices (LRIs) on DB-5-MS apolar column; ^b^ Linear Retention Indices based on literature [19].

## Data Availability

The original contributions presented in this study are included in the article/Supplementary Materials. Further inquiries can be directed to the corresponding authors.

## Supplementary Materials

The following supporting information can be downloaded at https://www.mdpi.com/article/10.3390/molecules30214300/s1: Figure S1: Total chromatogram and mass spectra of methylated fatty acids of *Nigella sativa* seed oil. Figure S2: Images of nuclei obtained with a confocal fluorescence microscope, stained with ethidium bromide (5 µg/mL). (A) control (B) cells treated with 1 mg/mL *NS* (C) cells treated with 2 mg/mL *NS* (D) cells treated with 4 mg/mL *NS*.
