# Supplementary material for: Analytical Determination of the Lipid Fraction of Nigella sativa Fatty Oil by GC and NMR Analysis and Evaluation of Its Cytotoxic and Antioxidant Activity
Source: Molecules. 2025 Nov 5;30(21):4300. doi: 10.3390/molecules30214300 (PMC12611037; doi:10.3390/molecules30214300)
Supplement: Supplementary file 1 [file molecules-30-04300-s001.zip › molecules-3938578-supplementary.pdf]

Supplementary Materials

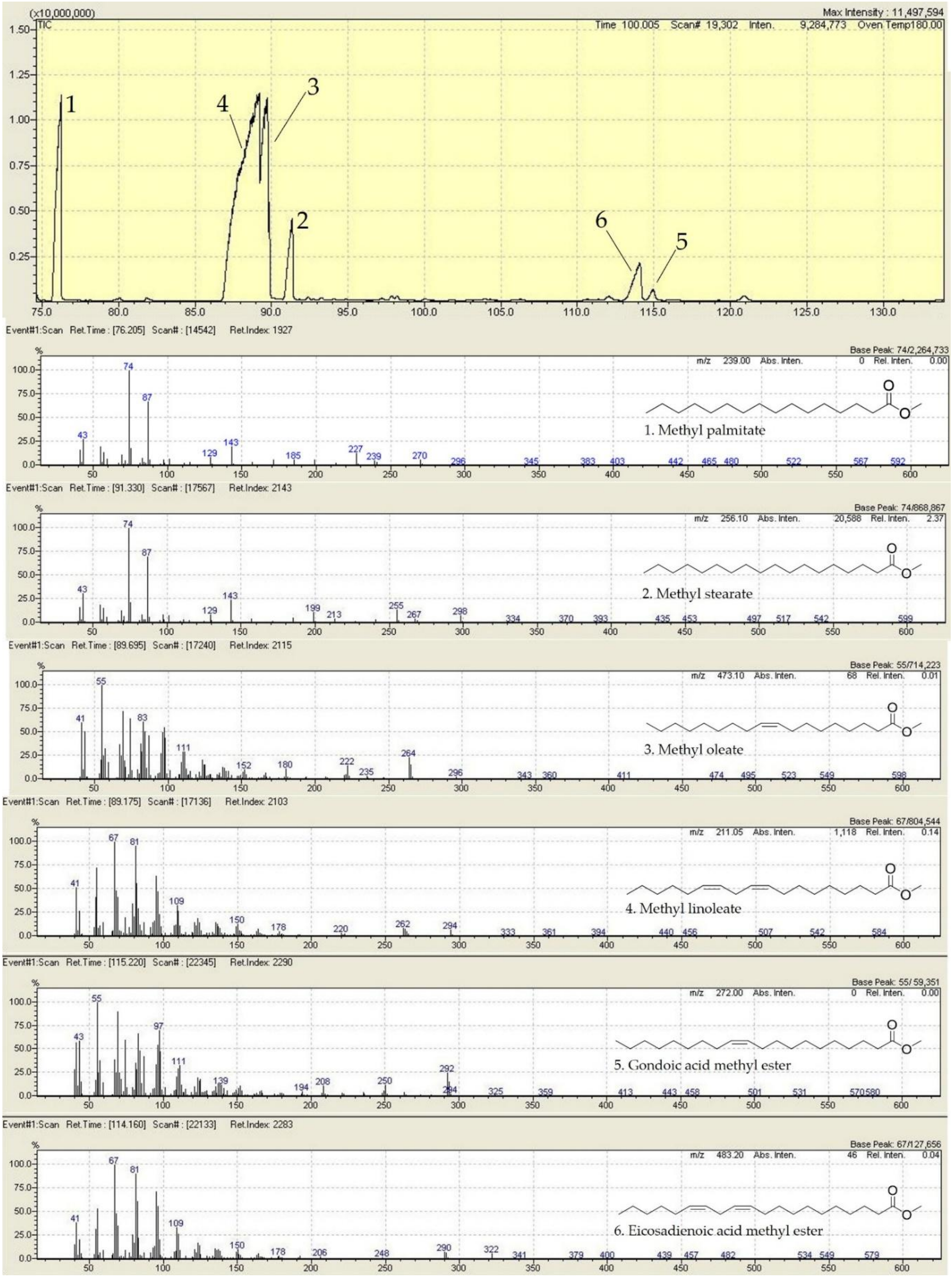

Figure S1. Total chromatogram and mass spectra of methylated fatty acids of *Nigella sativa* seed oil.

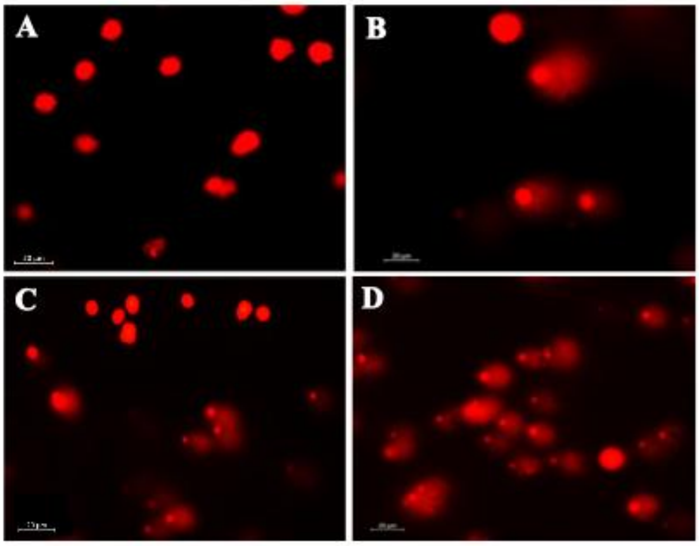

Figure S2. Images of nuclei obtained with a confocal fluorescence microscope, stained with ethidium bromide (5 μg/mL). (**A**) control (**B**) cells treated with 1 mg/mL *NS* (**C**) cells treated with 2 mg/mL *NS* (**D**) cells treated with 4 mg/ml *NS*.
